# Self-supervised monocular depth estimation for high field of view colonoscopy cameras

**DOI:** 10.3389/frobt.2023.1212525

**Published:** 2023-07-25

**Authors:** Alwyn Mathew, Ludovic Magerand, Emanuele Trucco, Luigi Manfredi

**Affiliations:** ^1^ Division of Imaging Science and Technology, School of Medicine, University of Dundee, Dundee, United Kingdom; ^2^ Discipline of Computing, School of Science and Engineering, University of Dundee, Dundee, United Kingdom

**Keywords:** colonoscopy, depth estimation, wide-angle camera, endorobot, navigation

## Abstract

Optical colonoscopy is the gold standard procedure to detect colorectal cancer, the fourth most common cancer in the United Kingdom. Up to 22%–28% of polyps can be missed during the procedure that is associated with interval cancer. A vision-based autonomous soft endorobot for colonoscopy can drastically improve the accuracy of the procedure by inspecting the colon more systematically with reduced discomfort. A three-dimensional understanding of the environment is essential for robot navigation and can also improve the adenoma detection rate. Monocular depth estimation with deep learning methods has progressed substantially, but collecting ground-truth depth maps remains a challenge as no 3D camera can be fitted to a standard colonoscope. This work addresses this issue by using a self-supervised monocular depth estimation model that directly learns depth from video sequences with view synthesis. In addition, our model accommodates wide field-of-view cameras typically used in colonoscopy and specific challenges such as deformable surfaces, specular lighting, non-Lambertian surfaces, and high occlusion. We performed qualitative analysis on a synthetic data set, a quantitative examination of the colonoscopy training model, and real colonoscopy videos in near real-time.

## 1 Introduction

Colorectal cancer (CRC) causes approximately 900,000 deaths worldwide (Bray et al., 2018) and around 16,800 deaths in the United Kingdom annually. A total of 42,900 new cases were reported in the UK in 2018, making CRC the third most common cancer, accounting for 11% of all new cancer cases. Optical colonoscopy is the gold standard procedure for CRC screening. However, its quality is strongly dependent on the gastroenterologist’s skill level (Baxter et al., 2009). Recent studies by le Clercq et al. (2014) have shown that around 60% of CRC cases are related to missed lesion detection from a previous colonoscopy procedure. Chromoendoscopy can increase the visibility of a lesion by sparing and rinsing the colon with a topical dye that increases the colonic wall surface contrast (Durr et al., 2014). However, this procedure requires twice as much time (Pohl et al., 2011), making it undesirable when compared to optical colonoscopy.

The recent introduction of computer-assisted technologies using artificial intelligence for colonoscopy has demonstrated promising results in polyp detection (Lee et al., 2020), size classification (Itoh et al., 2018), and colon coverage (Freedman et al., 2020). The field of robotics has also shown interest in new technologies for colonoscopy (Ciuti et al., 2020), capsules (Formosa et al., 2019), endorobots (Manfredi (2021, 2022)), meshworm robots (Bernth et al., 2017), soft robots (Manfredi et al., 2019), and autonomous robots (Kang et al., 2021). A colonoscope is a long, flexible tubular instrument, generally 12 mm in diameter with a single camera head, lights, irrigation, and an instrument channel port. The miniature camera and the lights allow visual inspection of the colon, and the irrigation port equipment with water helps clean the colon from residual stools. The instrument port enables the passage of surgical tools to remove tissue or polyps for further examination. The size of the colonoscope is restricted by the colon size; thus, the number of sensors to be included, such as three-dimensional (3D) cameras or other sensors, is limited. This has led researchers to investigate 3D mapping from images from a monocular camera inside the colon (Mahmood et al., 2018).

In recent years, monocular depth estimation has been studied with supervised learning (Nadeem and Kaufman, 2016), conditional random fields (Mahmood and Durr, 2018), generative adversarial networks (GANs) (Mahmood et al., 2018), conditional GANs (Rau et al., 2019), and self-supervised learning (Hwang et al., 2021). However, previous works had not considered that the colonoscope camera yields highly distorted images as it uses high field-of-view (FOV) cameras. Cheng et al. (2021) used synthetic data with ground-truth depth to train the baseline supervised depth model used in a self-supervised pipeline and used optical flow from a pre-trained flow network for temporal structural consistency. However, optical flow in a deformable environment like a colon is unreliable. In Bae et al. (2020), the training pipeline follows multiple steps: sparse depth from Structure from Motion (SfM), sparse depth used to train a depth network, pixel-wise embedding, and multi-view reconstruction, which makes it slower and unsuitable to run alongside a robot. The main contributions of the proposed work are:

1. Introduces self-supervised monocular depth estimation methods specifically designed for wide FOV colonoscopy cameras, addressing the challenges posed by the distorted and wide-angle nature of the images.
2. The proposed approach effectively handles low-texture surfaces in view synthesis by incorporating a validity mask, which successfully removes pixels affected by specular lighting, lens artifacts, and regions with zero optical flow, ensuring accurate depth estimation even in challenging areas of the colon.

## 2 Motivation

**FIGURE 1:**
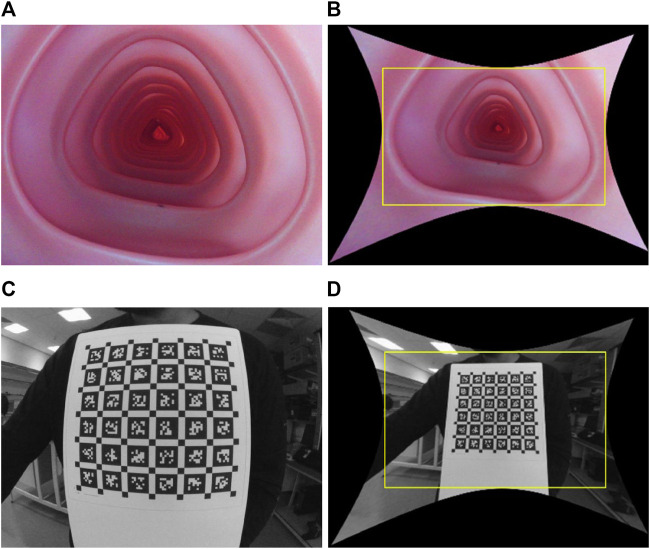
Illustration of wide FOV colonoscopy camera and its distortion: raw and rectified images, respectively, when the camera is placed inside the colonoscopy training model **(A)** and **(B)**, and in front of a calibration target **(C)** and **(D)**. The yellow box in **(B)** and **(D)** crops out the black pixels after undistortion, causing a loss of FOV.

Colonoscopy cameras generally have a wide FOV that enables faster diagnosis with complete coverage of the colon wall. The state-of-the-art colonoscopy devices have a 140°*−*170° FOV camera. As a result, these cameras suffer from high lens distortion, especially radial, as shown in Figure 1, but have not yet been studied sufficiently because most endoscopy depth estimation methods are trained on low FOV images or synthetic images without distortion.

### 2.1 Low FOV cameras

Low FOV cameras have *<*120^*◦*^ horizontal FOV lenses as shown in the UCL 2019 data set (Rau et al., 2019). The Brown–Conrady camera model (Kannala and Brandt, 2006) considers these lenses with both radial and tangential distortion. The UCL 2019 data set (Rau et al., 2019) contains synthetic images generated with the ideal pinhole camera approximation. Image distortion is prevalent in real images caused by varying lens magnifications along the increasing angular distance. Due to the imperfect alignment of the lens or sensors, distortion may get decentered. The Brown–Conrady (Kannala and Brandt, 2006) projection function $\pi \left(P,i\right):\;P\;\left(x,y,z\right)\;\to \;p\left(u,v\right)$ is defined as

$$x^{\prime }=\frac{x}{z}\;y^{\prime }=\frac{y}{z},$$

$$x^{\prime \prime }=x^{\prime }\left(1+k_{1}r^{2}+k_{2}r^{4}+k_{3}r^{6}\right)+2p_{1}x^{\prime }y^{\prime }+p_{2}\left(r^{2}+2x^{\prime 2}\right),$$

$$y^{\prime \prime }=y^{\prime }\left(1+k_{1}r^{2}+k_{2}r^{4}+k_{3}r^{6}\right)+p_{1}\left(r^{2}+2y^{\prime 2}\right)+2p_{2}x^{\prime }y^{\prime },$$

$$u=x^{\prime \prime }f_{x}+c_{x}\;v=y^{\prime \prime }f_{y}+c_{y},$$ (1)

where *P* is a 3D camera point with coordinates (*x, y, z*), *p* is a 2D image point with coordinates (*u, v*), and *r* ^2^ = *x* ^2^ + *y* ^2^. Camera parameters *i* consist of radial distortion coefficients (*k*_1_ *, k*_2_ *, k*_3_), tangential distortion coefficients (*p*_1_ *, p*_2_) of the lens, focal lengths (*f*_*x*_ *, f*_*y*_), and principal points (*c*_*x*_ *, c*_*y*_).

### 2.2 Wide FOV colonoscopy cameras

Omnidirectional camera model (Scaramuzza et al., 2006), unified camera model (UCM) (Geyer and Daniilidis, 2000), extended UCM (eUCM) (Khomutenko et al., 2015), or double sphere (DS) camera model (Usenko et al., 2018) can map wide FOV lenses. The omnidirectional camera model along with the pinhole radial distortion (Fryer and Brown, 1986) and Brown–Conrady (Kannala and Brandt, 2006) camera models falls in the high-order polynomial distortion family. These models require solving the root of a high-order polynomial during unprojection, which is computationally expensive. On the other hand, UCM, eUCM, and DS fall into a unified camera model family that is easy to compute and has a closed-form unprojection function. For briefness, we only describe the DS in this article, but this pipeline can be extended to all unified camera models. DS is modeled with six camera parameters *i*: *f*_*x*_, *f*_*y*_, *c*_*x*_, *c*_*y*_, *α*, and *ξ*.

In the study by Geyer and Daniilidis (2000), it was observed that the UCM effectively represents systems with parabolic, hyperbolic, elliptic, and planar mirrors. Furthermore, the UCM has been successfully employed for cameras equipped with fisheye lenses (Ying and Hu, 2004). However, it is important to acknowledge that the UCM may not provide an ideal match for most fisheye lenses, often necessitating the incorporation of an additional distortion model. The UCM projects a 3D point onto the unit sphere and then onto the image plane of the pinhole camera, which is shifted by *ξ* from the center of the unit sphere. The eUCM can be considered a generalization of the UCM, where the point is projected onto an ellipsoid symmetric around the *z*-axis using the coefficient *β*. Alternatively, the DS (Usenko et al., 2018) camera model is a more suitable choice for cameras with fisheye lenses. It offers a closed-form inverse and avoids computationally expensive trigonometric operations. In DS, a 3D point is projected onto two unit spheres with centers shifted by *ξ*. Then, the point is projected onto an image plane using a pinhole shifted by α. It corrects the image distortion that occurs when the camera’s image plane 1*−α* is not perfectly aligned with the object plane. The projection function *π*(*P*, *i*): *P*(*x*, *y*, *z*) *→ p* (*u*, *v*) is given as follows:

$$\pi \left(P,i\right)=\left[\begin{array}{c} f_{x}\frac{x}{x\;\alpha d_{2}+\left(1-\alpha \right)\left(\xi d_{1}+z\right)}\\ f_{y}\frac{y}{\alpha d_{2}+\left(1-\alpha \right)\left(\xi d_{1}+z\right)} \end{array}\right]+\left[\begin{array}{c} cx\\ cy \end{array}\right],$$ (2)

where

$$d_{1}=\sqrt{x^{2}+y^{2}+z^{2}}\;\;d_{2}=\sqrt{x^{2}+y^{2}+{\left(\xi d_{1}+z\right)}^{2}}.$$ (3)

The unprojection function $\pi^{-1}\left(p,i\right):\;p\left(u,v\right)\;\to \;P\;\left(x,y,z\right)$ is given as follows:

$$\pi^{-1}\left(p,i\right)=\frac{m_{z}\xi +\sqrt{m_{z}^{2}+\left(1-\xi^{2}\right)r^{2}}}{m_{z}^{2}+r^{2}}\;\left[\begin{array}{c} m_{x}\\ m_{y}\\ m_{z} \end{array}\right]-\left[\begin{array}{c} 0\\ 0\\ \xi \end{array}\right],$$ (4)

where

$$m_{x}=\frac{u-c_{x}}{f_{x}}\;m_{y}=\frac{u-c_{y}}{f_{y}}\;r^{2}=m_{x}^{2}+m_{y}^{2}.$$

$$m_{z}=\frac{1-\alpha^{2}r^{2}}{a\sqrt{1-\left(1-\left(2\alpha -1\right)r^{2}+1-\alpha \right)}}.$$ (5)

## 3 Methods

**FIGURE 2:**
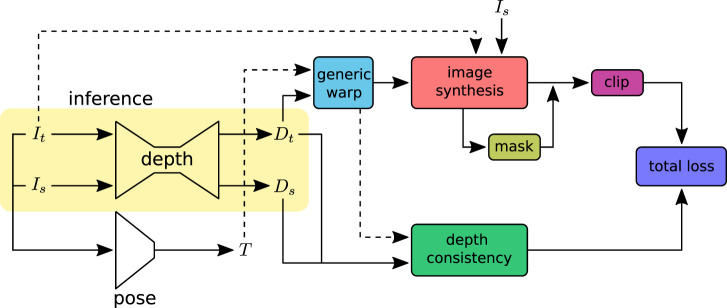
Self-supervised depth training pipeline: input images (*I*_*s*_, *I*_*t*_) are adjacent frames sampled from a video sequence. The *depth* network predicts independent per-pixel dense depth maps (*D*_*s*_, *D*_*t*_) from the source *I*_*s*_ and target *I*_*t*_ images, respectively. The *pose* network takes the concatenated image sequence (*I*_*s*_, *I*_*t*_) as input and predicts the rotation *R* and translation *t* that define the transformation *T* between the source and target images. The *warp* function takes the predicted depths and poses to warp the source image coordinates to the target or *vice versa*. The *image synthesis* projects the source image *I*_*s*_ to the target image *I*_*t*_ with the warped image coordinates. The synthesized images are compared with ground-truth images to construct the loss. The image synthesis loss outliers are removed using the *clip* function. Image pixels affected by specular lighting and violation of the Lambertian surface assumption are ignored in loss calculation with the valid *mask*. The *depth consistency* loss ensures that the depth is consistent in the sequence. At inference, only the depth network will be used.

In this work, we aim to estimate depth from a colonoscopy image stream via view synthesis. View synthesis enables us to train the depth estimation model in self-supervised mode. The depth network takes one image at a time, source *I*_*s*_ or target *I*_*t*_ image, and predicts the corresponding per-pixel depth maps. Target depth maps *D*_*t*_ from the depth network and *T*_*t→s*_ from pose estimation enable the reconstruction of the target image *I* ^ˆ^_*t*_ from the source image *I*_*s*_ with geometric projection. The pose network predicts the rigid transformation with six degrees of freedom. With a chosen camera model, the unprojection function *π* ^*−*1^ can map the target image coordinate *I*_*t*_(*p*) to 3D space, forming a 3D point cloud. An overview of the training pipeline is shown in Figure 2. The projection function *π* maps the 3D points to the target image coordinates. Section 2 describes the camera models for different lenses with varying lens distortions. For camera models that have to find the root of a high-order polynomial, a pre-calculated lookup table can be used for a computationally efficient unprojection operation.

### 3.1 Generic image synthesis

**FIGURE 3:**
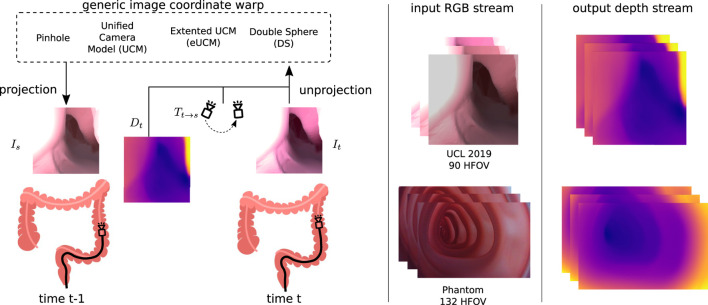
Generic image coordinate warp block: source *I*_*s*_ and target *I*_*t*_ images are captured at time *t* − 1 and *t*. The depth *D*_*t*_ predicted by the *depth* network along with the relative pose *T*_*t→s*_ from the source to target estimated by the *pose* network is used to synthesize the source image. The view synthesizing block allows the usage of various camera models that can handle cameras with higher lens distortion. We have demonstrated this use case on the UCL 2019 synthetic data set with a 90^*◦*^ horizontal FOV camera (no lens distortion) and on our colonoscopy training model data set with a 132^*◦*^ horizontal FOV camera (high radial lens distortion).

Irrespective of the camera models shown in Eq 1 or Eq 2-4, the generic image synthesis block can reconstruct the target image from source images with projection *π* and unprojection *π* ^*−*1^ functions, as shown in Figure 3. A point cloud *P*_*t*_ can be generated from the estimated depth *D*_*t*_ for the corresponding input image *I*_*t*_ as follows:

$$P_{t}=\pi^{-1}\left(pt,D_{t}\right),$$ (6)

where *p*_*t*_ is the set of image coordinates and *P*_*t*_ is the 3D point cloud corresponding to the target image *I*_*t*_. The relative pose *T*_*t→s*_ from the target to source image is estimated by the pose network. This relative pose can be used to transform the target point cloud into the source point cloud *P*_*s*_ = *T*_*t→s*_ *P*_*t*_. With the projection model *π*, the 3D point cloud *P*_*s*_ can be mapped to the source image coordinate *p*_*s*_ as follows. The target image can be synthesized by inverse warping the source image coordinates and sampling (Godard et al., 2017) with *ζ*.

$$p_{s}=\pi \left(T_{t\to s}\pi^{-1}\left(p_{t},D_{t}\right)\right).$$

$${\hat{I}}_{t}^{\;ij}=\zeta \left(I_{s}^{\;i^{\prime }j^{\prime }}\right).\;$$ (7)

The projected source image coordinate *p*_*s*_(*i* ^*′*^ *, j* ^*′*^) will be used to sample pixel values with the sampling function *ζ* to generate the target image ${\hat{I}}_{t}$ . The differentiable spatial transformer network (Jaderberg et al., 2015) is used to synthesize images with bilinear interpolation.

### 3.2 Image reconstruction loss

The photometric loss *L*_*v*_ between the reference frame and reconstructed frame acts as a self-supervised loss for the depth network. Following the previous works of Zhou et al. (2017), we used structural similarity (SSIM) (Wang et al., 2004) along with the least absolute deviation (L1) for the photometric loss with weight *η*. This leverages the ability of SSIM, denoted as S, to handle complex illumination changes and add robustness to outliers as follows:

$$L_{v}^{s}\left(I_{t}{\hat{I}}_{t}\right)=\frac{1}{\left|V\right|}\sum_{p\in V}\eta \frac{1-S\left(I_{t}\left(p\right),{\hat{I}}_{t}\left(p\right)\right)}{2}+\left(1-\eta \right)\left|I_{t}\;\left(p\right)-{\hat{I}}_{t}\left(p\right)\right|.$$ (8)

Here, *V* denotes valid pixels and *|V|* denotes its cardinal. Masking out pixels with *V* that violates non-zero optical flow and the Lambertian surface assumption avoid the propagation of corrupted gradients into the networks. The valid mask is computed as follows:

$$V=M_{ego}\;\cdot \;\left(\left|I_{t}\left(p\right)-{\hat{I}}_{t}\left(p\right)\right|<\;\left|I_{t}\left(p\right)-I_{s}\left(p\right)\right|\right).$$ (9)

Here, *M*_*ego*_ is a binary mask that initially marks all mapped pixels as valid. The pixels that appear to be static between the source and target images caused by the absence of ego motion, special lighting, lens artifacts, and low texture are then marked invalid. Parts of the photometric loss that violate the assumptions generate a great loss, yielding a large gradient that potentially worsens the performance. Following the study of Zhou et al. (2018), clipping the loss with *θ*, which is the *p*-th percentile of *L*_*v*_, removes the outlines as *L*_*v*_(*s*_*i*_) = *min*(*s*_*i*_,*θ*), where *s*_*i*_ is the *i*-th cost in *L*_*v*_.

### 3.3 Depth consistency loss

Depth consistency loss *L*_*c*_ (Bian et al., 2021) ensures depth maps *D*_*s*_ and *D*_*t*_ estimated by the depth network with the source and target images *I*_*s*_ and *I*_*t*_ that adhere to the same 3D scene. Minimizing this loss encourages depth consistency not only in the sequence but also to the entire sequence with overlapping image batches. Depth consistency loss can be defined as follows:

$$L_{c}^{s}\left(D_{s},D_{t}\right)=\frac{1}{\left|V\right|}\sum_{p\in V}{DC\;}^{s},\;$$

$$DC^{\;s}=\frac{\left|{\hat{D}}_{s}^{t}\;\left(p\right)-{\hat{D}}_{s}\left(p\right)\right|}{{\hat{D}}_{s}^{t}\;\left(p\right)-{\hat{D}}_{s}\left(p\right)},$$ (10)

where ${\hat{D}}_{s}^{t}$ is the projected depth of *D*_*t*_ with pose *T*_*t→s*_ that corresponds to the depth at image *I*_*s*_, but comparing ${\hat{D}}_{s}^{t}$ with *D*_*s*_ cannot be done as the projection does not lie in the grid of *D*_*s*_. Thus, ${\hat{D}}_{s}^{t}$ is generated using differentiable bilinear interpolation on *D*_*s*_ and compared with ${\hat{D}}^{t}$ . The inverse of *DC* will act as an occlusion mask *M*_*o*_ that can mask out occluded pixels from the source to the target view, mainly around the haustral folds of the colon. The image reconstruction loss *L*_*s*_ is reweighted with *M*_*o*_ mask as follows:

$$L_{v}^{s^{\prime }}=\frac{1}{\left|V\right|}\;\sum_{p\in V}M_{0}^{s}∙L_{v}^{s}.\;$$

$$M_{0}^{s}=1-DC^{s}.\;$$ (11)

### 3.4 Edge-guided smoothness loss

The depth maps are regularized with edge-guided smoothness loss as the homogeneous and low-texture regions do not induce information loss in image reconstruction. The smoothness loss is adapted from Wang et al. (2018) as follows:

$$L_{e}^{s}\left(I_{t},D_{t}\right)\sum_{p\in I}e^{-▽It\left(p\right)}▽D^{\prime }\left(p\right),$$ (12)

where ▽ denotes the first-order gradient and $D_{t}^{\prime }$ = (1*/D*_*t*_)*/* *mean*(1*/D*_*t*_) is the normalized inverse depth. To avoid the optimizer getting trapped in the local minima (Zhou et al., 2017; Godard et al., 2019), the depth estimation and loss are computed at multiple scales. The total loss is averaged over scales (s = 4) and image batches as follows:

$$L=\frac{1}{4}\sum_{s=1}^{4}\sigma_{1}L_{v}^{s^{\prime }}+\sigma_{2}L_{c}^{s}+\sigma_{3}L_{e}^{s}.$$ (13)

## 4 Experiments

In this work, all models are trained in the UCL 2019 synthetic (Rau et al., 2019) and colonoscopy training model data sets and evaluated on a variety of synthetic (Rau et al., 2019; Azagra et al., 2022) and real (Azagra et al., 2022; Ma et al., 2021) colonoscopy data.

### 4.1 Implementation details

The depth network is inspired by Godard et al. (2019), with ResNet18 (He et al., 2016) as the encoder and multiscale decoder that predicts the depth at four different scales. The pose network shares the same encoder architecture as the depth network, followed by four 2D convolution layers. The models are built using PyTorch (Paszke et al., 2017) with the Adam optimizer (Kingma and Ba, 2014) to minimize the training error. The models are trained with Nvidia A6000 with batch size 25 for 20 epochs at an initial learning rate of 0*.*0001 and halves after 15 epochs. The depth network sigmoid output *d* is scaled as *D* = 1*/*(*d ·* 1*/*(*d*_*max*_ − *d*_*min*_) + 1*/d*_*min*_), where *d*_*min*_ = 0*.*1 and *d*_*max*_ = 20*.*0 units corresponding to 0*.*1–20 cm. The network input image size of the UCL 2019 synthetic image (Rau et al., 2019) is 256 × 256 (height × width) and that of the CTM is 512 × 768. These hyperparameters are set empirically as follows: *η* = 0*.*85, *σ*_1_ = 1*.*0, *σ*_2_ = 0*.*5, and *σ*_3_ = 0*.*001. We perform horizontal flipping with 50% of the images in the UCL 2019 data set but not in the CTM data set because of the off-centered principal point of the wide-angle camera. We perform random brightness, contrast, saturation, and hue jitter within ±0.2, ±0.2, ±0.2, and ±0.1 range, respectively. For stability, the first epoch of the training is a warm-up run with minimum reprojection loss (Godard et al., 2019) when trained on the CTM, whereas no warm-up is used for the UCL 2019 synthetic data set. The depth estimation model runs at 10 FPS (frames per second) on the GPU at inference, making it suitable for robotic control.

#### 4.1.1 UCL 2019 synthetic data set

The UCL synthetic data (Rau et al., 2019) are generated based on human CT colonography with manual segmentation and meshing. The unity engine is used to simulate the RGB images with a virtual pinhole camera (90^*◦*^ FOV and two attached light sources) and their corresponding depth maps with a maximum range of 20 cm. The data set contains 15,776 RGB images and ground-truth depth maps, which are split into the 8:1:1 train, validation, and test sets. The data set is recorded as nine subsets with three lighting conditions and one of three different materials. The light sources vary in spot angle, range, color, and intensity, while the materials vary in color, reflectiveness, and smoothness.

#### 4.1.2 Colonoscopy training model data set

Our colon training model data set is recorded with an off-the-shelf full HD camera (1920 × 1,080, 30 Hz, 132^*◦*^ HFOV, 65^*◦*^ VFOV, 158^*◦*^ DFOV, FID 45-20-70, white ring light around camera) inside a plastic phantom used for training medical professionals. The phantom mimics the 1:1 anatomy of the human colon, such as the internal diameter, overall length, and haustral folds, simulating images from an optical colonoscopy. The camera is attached to a motorized shaft that is moved forward and backward while recording videos. The data set contains around 10,000 images split into an 8:2 train and validation set with varying internal and external light settings and does not provide ground-truth depth maps.

### 4.2 Quantitative study

The unique structural restriction of a real colon precludes the collection of ground-truth depth with a true depth sensor. Thus, quantitative depth evaluation can only be conducted on synthetic data such as UCL 2019, even though the current synthetic data generation is not realistic enough to mimic real colon surface properties. The UCL synthetic data contains some texture, such as blood veins and haustral folds. The proposed depth estimation is assessed on the UCL 2019 test data set with evaluation metrics by Eigen et al. (2014) and compared with the other state-of-the-art methods like SfMLearner (Zhou et al., 2017), Monodepth1 (Godard et al., 2017), DDVO (Wang et al., 2018), Monodepth2 (Godard et al., 2019), HR Depth (Lyu et al., 2021), AF-SfMLearner (Shao et al., 2021), and AF-SfMLearner2 (Shao et al., 2022) in Table 1.

**TABLE 1:** Quantitative study on proposed depth model on UCL 2019 synthetic data. Best values are in bold, and the second best values are underlined. *Abs Rel*, *Sq Rel*, *RMSE*, and *RMSE log* are in mm, and *δ <* 1.25^1^ *, δ <* 1.25^2^ *,* and *δ <* 1.25^3^ are in percentage.

Model	Abs Rel *↓*	Sq Rel *↓*	RMSE *↓*	RMSE log *↓*	*δ <* 1*.*25^1^ *↑*	*δ <* 1*.*25^2^ *↑*	*δ <* 1*.*25^3^ *↑*
SfMLearner (Zhou et al., 2017)	0.451	0.711	1.768	0.537	0.366	0.618	0.785
Monodepth1 (Godard et al., 2017)	0.444	0.752	1.755	0.531	0.371	0.625	0.790
DDVO (Wang et al., 2018)	0.452	0.772	1.768	0.538	0.366	0.618	0.785
Monodepth2 (Godard et al., 2019)	0.446	0.756	1.757	0.532	0.360	0.624	0.789
HR Depth (Lyu et al., 2021)	0.448	0.762	1.762	0.534	0.369	0.621	0.788
AF-SfMLearner (Shao et al., 2021)	0.387	0.618	1.648	0.480	0.420	0.686	0.831
AF-SfMLearner2 (Shao et al., 2022)	0.352	0.545	1.581	0.448	0.445	0.712	0.852
Our	**0.141**	**0.115**	**0.669**	**0.179**	**0.828**	**0.959**	**0.985**

### 4.3 Qualitative analysis

**FIGURE 4:**
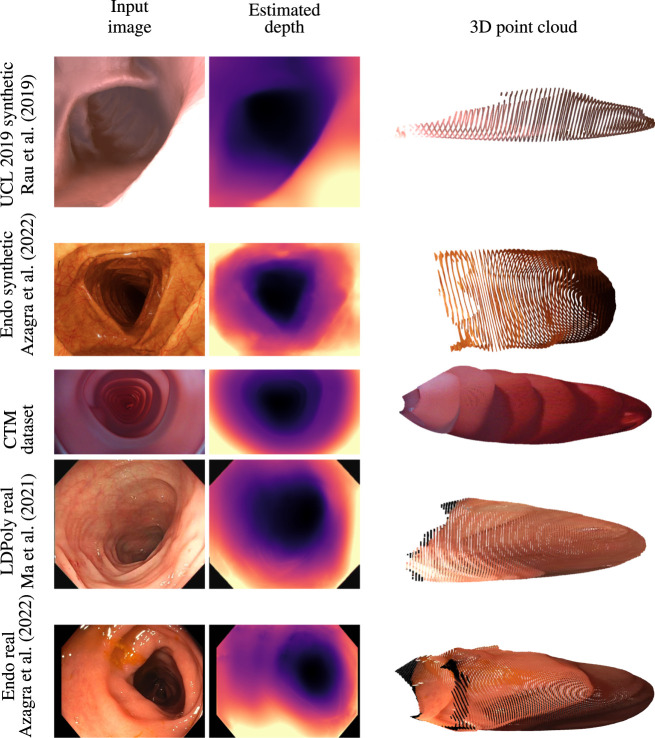
Qualitative analysis on synthetic data sets like UCL 2019 synthetic and EndoMapper synthetic data sets, CTM data set, and real colonoscopy images like LDPolypVideo and EndoMapper data set (top to bottom) (Azagra et al., 2022; Ma et al., 2021).

Quantitative depth evaluation of real colonoscopy data like EndoMapper (Azagra et al., 2022) or LDPolypVideo (Ma et al., 2021) is challenging. Much of the real characteristics of the colon, like reflectance of the surface and light diffusion of different tissue types, are hard to mimic in a simulator. In addition, a real colon is not rigid; the deformable property of the colon wall makes the view synthesis even more problematic. It was observed that the depth network was leaving holes in the depth maps when trained on the low-textured CTM with just image reconstruction loss. Low-textured regions often occur in the real colon wall, and handling these regions is critical for data-driven methods that rely on view synthesis. The valid mask described in Section 3.2 works well for removing pixels affected by specular lighting, lens artifacts, and zero optical flow regions. The data collection of the CTM was limited to the linear motion of the steady-speed motorized shaft inside the phantom on which the wide-angle camera was fixed. This limits the prediction of the depth network when introduced to real colon images with sharp turns. The depth consistency loss described in Section 3.3 helps the network to learn geometric consistency and avoid depth map flickering between frames. The depth network trained on the CTM learns to directly predict depth from highly distorted images with the help of the generic image synthesizer briefed in Section 3.1 that can handle a variety of camera models. The model trained with a pinhole camera was tested against an unseen EndoMapper synthetic data set (Azagra et al., 2022). The model trained on the wide-angle camera was tested against real colonoscopy data sets like EndoMapper (Azagra et al., 2022) and LDPolypVideo (Ma et al., 2021). It is to be noted that the model trained on a wide-angle camera performed better in real colonoscopy data sets that used wide FOV cameras than the model trained on synthetic data, as shown in Figure 4. The model trained on rectified or perfect pinhole camera images, as in UCL 2019, is suboptimal to the target colonoscopy camera, which is distorted and wide angled. If trained on raw images like in the CTM, the network learns distortion as part of the transfer function, and it is only weakly encoded and thus expected to be more robust.

### 4.4 Ablation study

Table 2 demonstrates the impact of each loss when trained with UCL synthetic data. The depth evaluation reflects the benefit of the valid mask in Section 3.2, which ignores pixels that do not contribute to the photometric loss. It is also found that clipping off high photometric loss improves model performance. Even though depth consistency in Section 3.3 leads to consistent depth in the sequence, it did not improve quantitative results but helped qualitatively on the CTM data set.

**TABLE 2:** Ablation study on UCL 2019 synthetic data. *View*, *Valid*, *Consistency*, and *Clip* represent image view synthesis, valid mask, depth consistency, and loss clipping, respectively. Best values are in bold and second best values are underlined. *Abs Rel*, *Sq Rel*, *RMSE*, and *RMSE log* are in mm, and *δ <* 1.25^1^ *, δ <* 1.25^2^ *,* and *δ <* 1.25^3^ are in percentage.

View	Valid	Consistency	Clip	Abs Rel *↓*	Sq Rel *↓*	RMSE *↓*	RMSE log *↓*	*δ <* 1*.*25^1^ *↑*	*δ <* 1*.*25^2^ *↑*	*δ <* 1*.*25^3^ *↑*
✓	✗	✗	✗	0.195	0.184	0.843	0.236	0.748	0.909	0.961
✓	✓	✗	✗	0.180	0.150	0.734	0.216	0.774	0.928	0.968
✓	✗	✗	✓	0.157	0.139	0.703	0.195	**0.803**	0.945	0.981
✓	✓	✗	✓	**0.141**	**0.115**	**0.669**	**0.179**	0.828	**0.959**	**0.985**
✓	✗	✓	✗	0.179	0.144	0.725	0.215	0.779	0.929	0.970
✓	✓	✓	✗	0.171	0.139	0.729	0.209	0.791	0.933	0.971
✓	✓	✓	✓	0.176	0.140	0.699	0.211	0.788	0.931	0.970

## 5 Conclusion

This work presents a self-supervised depth estimation model for wide-angle colonoscopy cameras. To the best of our knowledge, this is the first time data from a wide-angle colonoscopy camera is used to estimate depth from a video sequence. Most of the previous works had assumed a pinhole model for the colonoscopy camera, but real colonoscopy cameras have a wide FOV lens. Our network predicts depth directly on highly distorted raw images typical for such real cameras. The pipeline also focuses on handling texture regions in the images that generate low photometric loss and geometrically consistent depth estimation in the sequence. Our methods fill a gap in modeling wide FOV cameras and low-texture regions in colonoscopy imaging. We also achieved near-real-time prediction with the depth estimation models that allow us to operate alongside a colonoscopy robot.

A limitation of our work is that the UCL 2019 synthetic data set does not fully capture the complex surface properties and characteristics of real colon tissue. The absence of ground-truth depth maps for the CTM data set further limits the ability to quantitatively assess performance on this data set. Further exploration and evaluation of diverse and realistic data sets can enhance the generalizability and reliability of the proposed depth estimation method.

## Data Availability

The data sets presented in this study can be found in online repositories. The names of the repository/repositories and accession number(s) can be found in the article.
